# Culinary medicine course: qualitative assessment of an innovative pedagogical approach

**DOI:** 10.1017/S1368980025100414

**Published:** 2025-06-30

**Authors:** Maryline Vivion, Valérie Trottier, Michel Lucas

**Affiliations:** 1 Department of Social and Preventive Medicine, Faculty of Medicine, Université Laval, Québec, QC, Canada; 2 Centre Hospitalier Universitaire de Québec – Université Laval Research Centre, Québec, QC, Canada

**Keywords:** Nutrition education, Medical education, Qualitative research, Student feedback

## Abstract

**Objective:**

While nutrition plays a major role in health, medical students have generally not received adequate nutritional education, lack confidence in their nutritional knowledge and feel unqualified to offer nutrition advice to future patients. Culinary medicine programmes have been developed to address this gap and employ an active learning approach that integrates medical and nutritional learning with the acquisition of culinary competencies and skills. This study aimed to qualitatively evaluate the Université Laval culinary medicine course based on students’ experiences of the course structure, active learning approach and its influence on their lifestyle, clinical practice and future approach to nutrition as physicians.

**Design:**

Discussion groups were conducted. Thematic content analysis of discussion group data was performed.

**Setting:**

A first French-language culinary medicine course was developed and pilot tested at Université Laval. The curriculum of this course combined online training videos on medical and nutritional concepts, hands-on cooking sessions and the realisation of a collaborative project.

**Participants:**

Pre-clerkship medical students enrolled in the elective culinary medicine course at each pilot project semesters (fall 2022: *n* 12, winter 2023: *n* 12).

**Results:**

Students valued the course’s innovative active learning approach, noting improvements in their diet, nutrition and cooking knowledge, skills, self-efficacy and confidence. They also developed greater critical thinking regarding nutrition and recognised their role in collaborating with dietitians.

**Conclusion:**

The culinary medicine course demonstrated prospective benefits for medical students, potentially improving their personal and future patients’ health and the integration of nutrition into medical education and practice.

## Culinary medicine

Despite the major role of nutrition in health, medical students are not sufficiently trained or academically supported in this field^(1,2)^. As systematic review reported that many medical students lack confidence in their nutritional knowledge, have not received adequate education and feel unqualified to offer nutrition advice to future patients^(2)^.

Culinary medicine programmes have been developed to address this gap in nutrition training within medical curricula^(3–5)^. This innovative approach complements traditional medical nutrition education by integrating medical and nutritional learning with the acquisition of culinary competencies and skills. It employs an active learning approach that uses culinary arts as a tool to translate theoretical knowledge into practical, multisensory and engaging experiences^(6)^. Culinary medicine aims to enhance the nutritional knowledge and skills of medical students for the benefit of both themselves and their future patients. This approach is grounded in the concept of food agency, which envisions individuals as being empowered to act with consequence^(7)^.

## Culinary medicine course pilot-tested at Université Laval

To address a gap in medical nutrition training, the first French-language culinary medicine course was developed and tested as a pilot project at Université Laval. The elective course, offered to two cohorts of medical students, had the following objectives: (1) improve knowledge of empirical evidence in four fields: medical, nutritional, culinary, organoleptic and flavor sciences; (2) develop students’ confidence and interest in nutrition and cooking; (3) enhance their practical culinary skills and multisensory experimentation; (4) improve their diet and relationship with food and (5) develop their food agency (self-efficacy and capacities to act). A quantitative study assessing the impact of the course reported a significant increase in students’ confidence regarding their nutritional knowledge and counselling skills, as well as an improvement in their food agency score^(1)^.

## Qualitative evaluation of the culinary medicine course tested at Université Laval

To complement the quantitative evaluation and recognise the value of the diverse perspectives offered by a qualitative assessment of the innovative pedagogical approach in culinary medicine^(8–15)^, this study aims to qualitatively evaluate the Université Laval culinary medicine course based on students’ experiences. Specifically, the objectives are to assess: (1) the course organisation, (2) students’ appreciation of the active learning approach, (3) the course’s impact on students’ lifestyle habits and (4) the course’s influence on students’ practice and vision of nutrition as future physicians.

## Methods

### Background

The culinary medicine course was offered as a pilot project at Université Laval for two semesters: fall 2022 (from September 6 to December 16) and winter 2023 (from January 10 to April 21), with minor adjustments to the course organisation. Each semester, the three distinct components of the course were featured:

The first component consists of online training videos on medical and nutritional concepts, covering ten major themes^(1)^ (see Table 1) and presented by various specialists^(1)^. The course also includes presentations on organoleptic qualities and flavours science. In total, twenty-six online videos provide approximately 23h of training. During the first semester, each student was required to watch all the videos. In the second semester, for each of the ten themes, a different team of two students was assigned to view the related videos and summarise them in a 10-minute presentation, delivered during the mealtime of the cooking classes (the second component of the course).

**Table 1.** Themes covered by online training videos and types of cuisine experimented in classes

Online training videos	Cooking classes
Introduction to culinary medicine	Mediterranean cuisine
Food marketing and communication	Vegetarian cuisine
Popular diets and weight	Planned cooking (batch cooking)/World cuisine
Social health inequalities and food insecurity	Plant-based cuisine
Women’s health	Allergen-free cuisine (gluten, dairy products, eggs)
Men’s health	Japanese cuisine
Exploring food allergies and the microbiota	Indian/ayurvedic cuisine
Elderly health	Fermentation and budget-friendly cuisine
Eating behaviours and a healthy relationship with food	
Literacy and food agency	

The second component consists of hands-on cooking classes and food tasting experiences, focusing on culinary techniques, cooking practice and multisensory culinary experiments. These elements of culinary art are explored in eight classes featuring various types of cuisine (Table 1). Each class, lasting approximately four hours^(1)^, includes shared meals from the dishes prepared during class, discussions of patient counselling scenarios and presentations of online video summaries. Classes were held in person at the École Hôtelière de la Capitale (ÉHC) in Québec City, Canada, which has specialised in training cooks and chefs for several decades. Students were guided by three instructors, including two qualified chefs, Eric Villain and Dr Michel Lucas and an experienced clinical physician, Dr. Sylvie Dodin. Chef Eric Villain, who has taught at ÉHC for nearly 25 years, brings extensive experience. In each class, students worked in pairs to prepare three different recipes, with two teams facing each other. The chefs prepare two or three additional recipes to expand the discovery and tasting experience related to the class theme, allowing students to explore a total of about five or more recipes per class. Overall, the ÉHC-based classes provided approximately 32 training hours.

The third component mainly involves the completion of a collaborative team project during the semester, focusing on activities related to dialogue, health promotion and social responsibility. Students were required to organise, seek funding for and carry out a project addressing a problem related to a nutrition-cooking topic presented by a partner at the beginning of the semester.

### Participants and recruitment

Participants were pre-clerkship students (first to third year) enrolled in the Université Laval Medicine programme, who took part in the elective culinary medicine course offered as a pilot project during the fall 2022 semester (*n*=12) and the winter 2023 semester (*n*=12). The majority of students were in the second year (53·6 %), followed by the third year (42·9 %) and first year (3·6 %). These students were invited by the office of undergraduate medical education at Université Laval to enroll on the course. As the number of applications surpassed the maximum capacity of twelve places per session, twelve students were selected by drawing lots. All students enrolled in the pilot course consented to participate in the qualitative evaluation.

### Data collection

A semi-structured discussion guide, developed by the research team and informed by literature on innovative teaching practices^(16)^ and study objectives, was validated by co-researchers. The guide covered the following themes: (1) overall course organisation, (2) appreciation of the active learning approach, (3) the course’s impact on students’ lifestyle habits and (4) the course’s influence on future doctors’ practice and perspective on nutrition. Discussion groups were conducted by Maryline Vivion, an experienced researcher in qualitative assessments and held in French, in person at Université Laval at the end of each semester. Special attention was given to ensuring that all students had the opportunity to express their opinions. The discussion groups, lasting approximately 90 min each, were audio recorded.

### Data analysis

The audio recordings of the discussion groups were selectively transcribed verbatim in French. Thematic content analysis was carried out using Excel spreadsheets, a process that involves categorising the content into themes representative of the research objectives^(17)^. The analysis was guided by the study objectives, and participant’s quotes could be associated with multiple themes. Representative quotes were selected, translated into English and validated by all researchers.

## Results

### Course organisation

#### Component 1: Online training videos

Students appreciated the video format, as it differed from the usual text-based courses in the medical programme: ‘[…] I really liked that it was in video format and not in reading format, […] you know, we end up reading it, but sometimes we read lines without really absorbing them. But to hear it, having it explained to us […] I found that really interesting, a great format!’

The students also found the video content engaging and relevant. The diversity of topics and presenters allowed exposure to multiple perspectives and encouraged reflection: ‘[…] the conferences each week were really…let’s say, quite different opinions. […] So it was fun to see different sides of the coin and reflect on them afterward. And it was also nice that there were different presenters each week for various topics, so it wasn’t just one perspective on things.’

However, students from the first cohort, who were required to view all videos, found them too long and numerous, leading to a decline in their attention and motivation. In contrast, where students from the second cohort worked in pairs to summarise videos, they appreciated the reduced workload. This allowed them to focus on key points. Additionally, preparing video summaries helped students develop skills in scientific vulgarisation and synthesis. The meal-sharing period during the cooking classes was seen as a favourable setting for presenting these summaries.

#### Component 2: Hands-on cooking class

Students expressed high satisfaction with the organisation of the hands-on cooking classes. They appreciated the efficient planning and timing of meal preparation: ‘It’s really well organised. I realised that […] it’s really more complicated than you think to set schedules, then planning steps for recipes, making sure we’re going to finish on time […]’ Most students enjoyed the variety of recipes they were able to cook and taste, including food they hadn’t prepared themselves.

#### Component 3: Collaborative team project

Since the students were responsible for raising the funds needed to execute their collaborative team project, many reported facing difficulties with this task: ‘It’s just that the financial stress associated with the project, we found it difficult, and like, I thought it was more relevant to student engagement than relevant to the course. But I really liked the fact that we had to do a project on social responsibility […]’ Some students also noted that the project created an excessive workload: ‘I was like ‘woah’ we’re at the end of the session, you know, we’ve got lots of other things on our schedule too […]’ Based on their feedback, the organisation of the collaborative team project would benefit from adjustments and a reduction in workload.

### Appreciation of the active learning approach

#### Response to the new approach

The active learning approach was highly appreciated by students from both cohorts. They found it enriching, relevant and refreshing to engage in hands-on learning rather than passive, lecture-based courses: ‘[…] It’s not really applicable to all courses, but for this type of course, I think it’s very relevant and the right way to do it. It’s much more active and proactive at the same time, for learning about good lifestyle habits, rather than… just learning the material. We’re more involved in it, in the learning process, than if we were just being taught concepts about nutrition.’

Students also reported becoming more comfortable in the active learning cooking environment. During the first class of hands-on cooking at ÉHC, an institution with professional facilities designed to train cooks and chefs, students from both cohorts initially felt unsettled and confused. They had to quickly adapt to many new aspects, such as the organisation of the kitchen, the use of culinary equipment, unfamiliar vocabulary, new food items and the instructions to follow. However, this initial confusion quickly dissipated in subsequent classes. As students progressed, they reported gaining confidence and developing more efficient workflows.

#### Interactions between students

Cooking in pairs was highly appreciated, with the kitchen layout facilitating collaboration and support between teams. Students reported that working in pairs helped them improve their teamwork, coordination and organisational skills: ‘[…] And that’s the kind of learning that we don’t really get in lectures – organising ourselves with someone else, beyond just our own studies – so learning to organise as a pair is something that will probably be useful later on too.’ Beyond nutrition and culinary skills, students consistently described learning experiences related to teamwork, communication and organisation. These are essential professional competencies. The structure of the course – particularly the paired cooking and team-based problem-solving – appeared to reinforce these skills in an applied, meaningful context. This highlights the broader educational value of culinary medicine in supporting the development of key interpersonal and organisational abilities.

Working in teams in the kitchen also encouraged discussions about topics covered in class, allowing different viewpoints to be heard and expressed. This teamwork further facilitated conversations on more sensitive subjects: ‘These are topics you can’t easily discuss in a 250-person lecture hall as you could around the table, with everyone’s opinions complement or sometimes even oppose each other a bit. But at least you get to see different points of view and different ways of seeing things, to make connections between them. And being in a small group made it easier to feel comfortable speaking up.’

Sharing and eating the meals together fostered discussions and made the students feel more comfortable with one another. It highlighted the importance of bonding over a shared meal and provided an opportunity to discuss about more personal topics, which was considered valuable.

In addition to the development of clinical and interpersonal skills, the course fostered a learning environment characterised by psychological safety and trust. The small-group, team-based structure encouraged open dialogue and peer support, allowing students to express differing viewpoints and engage in meaningful conversations – including on sensitive topics. Sharing meals further reinforced social connection and comfort, creating a space that supports both personal and professional growth in ways not typically possible in large lecture settings.

#### Interactions between instructors and students

Students from both cohorts also appreciated their interactions with the instructors. The friendly atmosphere in the kitchen relieved the pressure to perform, which is often felt in other medical courses: ‘I think it was nice, for once, not to have the pressure to perform. I think it’s been a topic in recent years—academic performance and performance anxiety, and the pressure to perform, especially in programmes like medicine.’

A sense of trust prevailed between the instructors and students, with students feeling comfortable asking questions, knowing the instructors were there to support their progress. The patience, attentiveness, understanding and flexibility of the instructors were also appreciated by students. ‘And also, I really liked that we could ask questions without being afraid of bothering the professors. Even if we made mistakes in the kitchen, because [laughs] […]. Even if we made mistakes, they didn’t get upset; they always had ways to adjust the recipe. […] So I really liked that. We felt confident with the professors, knowing they were there to teach us and help us.’

Students appreciated that the teaching was adapted to their progress throughout the hands-on cooking classes. For instance, as the semester advanced, demonstrations became shorter, reflecting the students’ growing understanding and autonomy. The personalised instruction in small cohorts further enhanced their positive experience of the culinary medicine course.

### The course impact on students’ lifestyle habits

#### Confidence, interest and self-efficacy regarding nutrition and cooking

Students from both cohorts reported that the culinary medicine course significantly boosted their confidence and autonomy in everyday cooking. They now feel more capable of following recipes and preparing meals that they would have previously bought pre-made. Students realised that learning new techniques and preparing unfamiliar dishes is often simpler than it appears: ‘It’s like some barriers have been broken down, and now we’re able to say, ‘Oh yeah, we know how to do that’, and we can do it.’

The course also encouraged students to engage in culinary discovery and exploration. With their new-found confidence, they now venture to incorporate a wider range of new foods and dishes into their diets. The extensive exposure to different flavours, recipes, food types and cultural influences presented in class inspired students to seek out new recipes and become more open to experimenting with different types of food.

The new knowledge and experience students gained during the course also encouraged them to improvise while cooking. With newly developed skills in executing recipes and using basic techniques, they now feel confident experimenting with and adapting recipes to suit their own tastes and needs: ‘I feel like I’m more capable of stepping outside the framework of a given recipe and improvising an improvement based on what we learned from this course.’

#### Social dimension, pleasure and sharing

Students also began to experience more pleasure in cooking and placed greater value on mealtimes. They now view meal preparation not as a task to be completed, but as an enjoyable activity worth dedicating time to, even amid their busy schedules.

They also recognised the importance of taking the time to eat without distraction, appreciating food for its true value and sharing the experience with others: ‘We also had the chance to explore some interesting modules on organoleptic qualities [videos], which gave us some insights into how to better taste different foods, appreciate their aromas, etc. […] I think we really developed our skills in this area, learning to appreciate food, to enjoy our meal, and to focus on eating without doing other things at the same time.’ Another student expressed: ‘[…] the meal part reminds us to take the time to stop, eat and appreciate the food, and do nothing else but eat in good company.’

#### Diet and critical thinking

After completing the culinary medicine course, students reported feeling more aware of the importance of healthy eating and expressed a desire to share their knowledge on the subject: ‘[…] there are also the modules [videos] that made me more aware of the importance of having a better diet. […] And that really stuck with me because we talked about it several times […]. So, it allowed me to talk about it with my family and discuss it as well, because it leads to discussions, not just among us, but also at home. We want to pass on the knowledge we’ve learned to others.’

Another important outcome was the development of critical thinking around diet, nutrition information and food systems. Students became more aware of the influence of commercialisation and conflicting messages in the nutrition space, and they reported feeling better equipped to evaluate such information: ‘Well, personally, I think that after seeing all these different positions that people can take, you put things into perspective a bit more […] I can spot bullshit now! [laughs] […] Because, you know, we’re vulnerable when it comes to food; we want to eat well, it’s natural, and unfortunately, there are people who take advantage of that.’ This ability to think critically about diet-related content is a core skill with wide applicability across medical practice, especially in an era where misinformation is prevalent and patients often seek guidance from their physicians.

### The course impact on students’ practice and vision of nutrition as future physicians

#### Inputs to the practice of medicine

According to students, when discussing diet with a patient during consultation, physicians trained in culinary medicine will be better equipped and more confident in offering basic nutritional recommendations based on the knowledge gained through this course: ‘[…] I think this course is really enriching […] it really equipped me with statistics and evidence-based, clinical data to give advice to patients and not feel confused. […] I feel that now, if a patient asks me, for example, ‘what do you mean by healthy lifestyle habits?’ or ‘what should I eat?’, I can at least give some basic advice and answer their main questions.’ According to another student: ‘What I took away is that it’s really important to equip students with knowledge about nutrition. With all the food-related confusion out there, it’s clear that we don’t have formal training in nutrition […] But it would be really fundamental to give medical students basic training in nutrition. I find it extremely important, especially because most cardiovascular diseases, […], etc., are primarily caused by diet. We really need to do something about that.’

Medical students will also be in a better position to understand the complexity or urgency of dietary issues that patients may face: ‘Well, I think we’ll be a bit better equipped to handle more urgent cases, or when there are more red flags. Let’s say, with eating disorders or more serious cases like diabetes and inflammatory diseases.’

The culinary medicine course also heightened students’ awareness of the importance of listening, openness and empathy, as well as the need to individualise their recommendations during consultations with future patients: ‘[…] you really have to individualise each situation, each person, considering their culture, habits and external influences. […] I think it’s really about individualising our approach and staying open to the person’s background and the opportunities they have to make change and add to their life.’

Students from both cohorts noted that what they learned in the course will help them convey to future patients that preparing, eating and sharing healthier meals, in addition to promoting physical health, can also support mental well-being.

#### The role of physicians *v.* dietitians

Students acknowledged that their nutrition knowledge is limited and not on par with that of a nutrition professional. However, they feel equipped to refer patients to a dietitian for further consultation and follow-up. Given the current lack of access to dietitians for the Québec population, physicians, as the first point of contact in healthcare, could help fill this gap while patients await consultation with a dietitian: ‘I think we’re all aware that we’ll never replace [a dietitian]… there are people who study four years just for that! But we also need to be aware of the current situation, where there is a shortage. The fact remains that doctors are often the first line of healthcare and often the first health professional a person sees is a doctor. So, we have no choice but to at least have… a basic knowledge in other fields. This doesn’t replace a nutrition referral for someone who’s diabetic, or someone with an intestinal issue […] being somewhat equipped in other areas of healthcare, is a necessity.’

## Discussion

This study provides a qualitative assessment of the first French-language culinary medicine course from the students’ perspective. While often perceived as a cooking class with added nutrition content, this course fostered learning far beyond culinary skills. Students demonstrated growth across multiple domains, including teamwork, communication, organisational skills, critical thinking and psychological safety. They engaged with complex issues such as nutrition misinformation, food industry influence and health behaviour change – skills that are highly relevant to medical practice. This broader impact reflects the deeper nature of cooking itself. As Amy Trubek and colleagues argue, cooking is not just a set of technical skills, but a dynamic, socially and culturally embedded competence^(7)^. It involves learning through repetition, experimentation and adaptation—what Tim Ingold describes as a pragmatic skill shaped by material tools, embodied practice and context^(18)^. Culinary medicine, then, becomes a powerful space for students to engage in experiential learning that is not only technical or nutritional but also deeply human, reflective and professionally formative.

This course was very well received by the two cohorts who participated in this pilot project, which is in line with other qualitative and quantitative studies reporting high levels of acceptability and satisfaction among culinary medicine students^(9,14,15)^. The course’s success was largely attributed to its effective organisation and active learning approach. One of the most established and widely implemented culinary medicine programmes is the CHOP programme (Cooking for Health Optimisation with Patients) at Tulane University’s Goldring Center for Culinary Medicine^(19,20)^. CHOP is a shorter course (32 h in eight 4-hour modules) focused on the Mediterranean diet and a nutrients-based framework. Université Laval culinary medicine course is longer (55 h over 15 weeks), combining 32 h of hands-on cooking with 23 h of broader nutrition education. By comparison, the course in this study delivers a particularly high ‘dose’ of training, with extensive didactic/video content and experiential kitchen-based learning. This level of exposure may contribute to shifts in learner attitudes and behaviours, though the amount and format of training required to produce these changes warrants further investigation.

Adjustments made to the second semester’s online video component were particularly beneficial, as students appreciated the reduced training time and workload – factors that can be barriers to the implementing of culinary medicine course^(12)^ in an already crowded medical curriculum^(2)^. This iterative adaptation between cohorts reflects the agility of the course leadership and underscores the importance of responsiveness in educational innovation. Flexibility and maintaining realistic goals were also identified as key factors for the success of such course^(12)^. Active learning approach in culinary medicine is highly appreciated^(10,13)^, preferred over traditional methods^(9)^ and linked to greater student satisfaction and engagement^(14)^. This approach is considered more effective than traditional clinical nutrition education in improving students’ nutrition knowledge and skills^(19–21)^. Active learning approaches are more effective in enhancing medical students’ clinical skills^(22)^ and improving STEM (science, technology, engineering and mathematics) students’ performance^(23)^, supporting the effectiveness of these methods. Our findings align with these studies, demonstrating that the active learning approach helped culinary medicine students feel more engaged in acquiring nutritional knowledge and cooking skills, allowing them to apply^(11,24)^ and consolidate their learning^(9)^. Additionally, the active approach encouraged teamwork^(12)^, fostered discussions and built camaraderie among peers^(25)^, which was highly appreciated by the students^(8,10)^. Most students did not know each other personally before enrolling in the culinary medicine course and would not have developed these interpersonal connections without the course. The pleasant atmosphere during meal preparation and tasting, along with trusting relationships with instructors, allowed students to grow in an environment free of pressure and stress^(25)^, further enhancing their positive experience with the active learning approach.

### The course’s impact on students’ lifestyle habits

The positive impacts of the course on medical students’ personal health and social aspects are numerous. First, the course significantly enhanced students’ confidence, interest and self-efficacy in nutrition and cooking. In line with the course objectives, students reported acquiring valuable nutritional knowledge, as highlighted in the literature^(8,9,11)^. The multisensory experiences offered by the course motivated students to deepen their food-related knowledge. Students also reported greater confidence in their nutritional understanding^(1,15)^, as well as improved critical thinking when assessing nutrition-related information^(10)^. Additionally, students noted significant improvements in their cooking confidence^(8,10,14,26)^ and abilities^(14,27)^ alongside a growing interest in culinary exploration^(8,10)^. They reported feeling more comfortable cooking new foods or dishes, following recipes and improvising, which contributed to their food self-efficacy^(1,8)^. Second, as anticipated, students also reported improvements in their own dietary intake^(1,8,14,15,19,20,27)^. The active learning approach of the culinary medicine course proved more effective than traditional medical nutrition education in improving the quality of students’ diets^(19–21)^, motivating them to make positive dietary changes for themselves, their loved ones and their future patients^(11)^. Finally, the social dimension of cooking and eating, emphasised throughout the course, was highly appreciated by students. Nutrition, in this context, stood out from the rest of the medical curriculum by integrating social values alongside physiological and biochemical principles^(13)^. This social aspect of culinary medicine further motivated positive changes in eating habits^(10)^. Students also reported finding pleasure in cooking, tasting and sharing healthier meals, which is linked to improved dietary habits^(28)^. They expressed a strong desire to share nutrition-related knowledge and the joy of healthy eating with those around them, including their patients. The collaborative team project provided students with an opportunity to share and apply their knowledge, reinforcing their confidences and desire to serve^(1)^. It not only benefited their academic learning but also helped develop communication, teaching and counselling skills in real-life situations, while deepening their understanding of public health issues^(14,24)^.

### The course’s impact on encouraging and fostering better food agency among students

The development of food agency among the medical students participating in the culinary medicine course is a critical outcome, enhancing both their personal and professional lives. In line with Ahearn’s broad definition of agency as a ‘socioculturally mediated capacity to act’^(29)^, the course empowers students to take an active role in their food choices. This empowerment stems from the course’s hands-on approach, enabling students to translate theoretical knowledge into practical skills, such as improvising in the kitchen, critically assessing nutrition information and adapting recipes to fit individual needs. As Merlan notes, ‘agency represents a “capacity to act consequentially in circumstances”^(30)^, and this was evident as students reported a heightened sense of control over their dietary choices and a greater willingness to experiment with new ingredients and cooking techniques.

This evolution in students’ food agency not only improved their personal health habits but also prepared them to guide future patients with evidence-based nutritional advice. Ortner’s^(31)^ concept of ‘agency of intention’ and ‘agency of power’ is relevant here, as the students’ newly acquired skills provide them with the ability to act, even when faced with obstacles. The collaborative nature of the course fostered a supportive environment, where students could share experiences, reinforcing their agency in making informed, health-conscious food choices.

In essence, drawing from Ingold’s perspective that cooking is a skilled practice shaped by the interplay of repeated activity, material objects and the acting subject^(18)^, the culinary medicine course cultivated a strong sense of food agency. This agency empowers students to act decisively and knowledgeably in both their personal and professional culinary practices, equipping them to navigate and shape their food environment with confidence and intention^(7)^.

### The course impact on students’ practice and vision of nutrition as future physicians

According to the literature, while most medical students believe that physicians have a role to play in nutrition care^(32)^ and that discussing dietary choices with patients is essential, only a minority feel comfortable doing so^(26)^. In line with another qualitative study^(14)^, our study addresses this gap, demonstrating that students believe they will be better prepared and more confident in offering basic nutrition recommendations to patients based on the knowledge gained from the culinary medicine course. Students reported that the training would equip them to provide evidence-based nutrition advice, adopt a more open and receptive attitude, individualise their recommendations and promote a gradual, pleasurable approach to improving patients’ diet. However, they also recognise the limitations of physicians’ nutrition expertise and emphasised the importance of referring patients to dietitians for more complex cases or further follow-up. Our study indicates that culinary medicine training could help physicians better identify patients in need of dietary referrals^(12,14)^, addressing the gap in current practice where physicians, despite the benefits, infrequently refer patients to dietitians and could improve their referral skills^(33)^. Given the current limited access to dietitians^(14)^, physicians could help bridge the gap by offering basic nutritional advice while patients await consultations. Such advice could play a preventive role in managing nutrition-related diseases, while also motivating patients to take their diet more seriously and not trivialise its importance in overall health. Moreover, physicians who practice healthy lifestyle habits, as promoted in culinary medicine, are perceived as more credible and motivating to their patients^(34)^ and are more likely to counsel them on lifestyle changes^(35)^. Developing interdisciplinary skills between physicians, dieticians and other health care professionals is also seen as essential for optimising healthcare delivery^(36)^. Students in this study recognised the importance of collaboration between physicians and dietitians and culinary medicine’s interdisciplinary nature fosters this collaboration, promoting interdisciplinary teamwork^(36)^.

The presence and engagement of professors also played a key role in shaping students’ perception of the course. Beyond delivering content, professors served as role models, demonstrating that nutrition is an essential part of clinical care. Their visible commitment to the subject helped legitimise its importance within the medical curriculum. As highlighted in the literature, such role modelling can influence students’ professional identity formation and signal that integrating nutrition into patient care is both relevant and expected in clinical practice^(37)^.

### Limitations

This study has a few limitations. Students’ perceptions were only captured at the end of culinary medicine training, with no prior qualitative assessment, making it difficult to observe changes in the students’ viewpoints over time. Another limitation is the potential for recall and social desirability biases, which can influence participants’ responses in qualitative research. These biases were minimised by having the discussion groups led by a researcher experienced in qualitative methods. However, demographic data and possible disparities between the two cohorts of students were not reported. As with most qualitative studies, the small sample size in this research is not meant to be representative of medical students and does not allow for generalisation of the findings. Lastly, it would be valuable to conduct longer-term evaluations to assess whether the positive impacts on students’ knowledge and skills related to nutrition are maintained over time and whether these improvements translate into enhanced patient counselling and well-being.

### Conclusions

This qualitative research complements the quantitative analysis of outcomes^(1)^ among medical students from the two cohorts who participated in the first French-language culinary medicine course. Findings from this study show that students greatly appreciated the innovative, active teaching approach of the course. Beyond enhancing their knowledge, confidence, interest and skills in nutrition and cooking, the course positively influenced their personal habits, particularly in terms of finding joy in cooking and sharing healthy meals, leading to improvements in their own diets. Students also reported a greater understanding of the importance of nutrition counselling and collaboration with dietitians in medical practice. Therefore, the culinary medicine course has the potential to benefit medical students, their future patients and the teaching and practice of medicine as a whole.

The findings from this study can inform the development of future culinary medicine courses aimed at improving nutrition education among physicians, ultimately contributing to a stronger healthcare system and better population public health outcomes.
